# Update on Oblique Flankplasty: Easily Executed, Long-Lasting, Integral Component of Total Body Lift Surgery

**DOI:** 10.1093/asj/sjad323

**Published:** 2023-10-04

**Authors:** Dennis J Hurwitz, Armando A Davila

## Abstract

**Background:**

Twenty years ago, coordinated aesthetic surgery for laxity and lipodystrophy after massive weight loss (MWL), so-called total body lift surgery (TBL), encompassed circumferential hip hugging transverse lower body lift (LBL) with possible buttock auto-augmentation, and a transverse bra line upper body lift (UBL) with breast reshaping. Brachioplasty and vertical thighplasty were often included. Disappointing aesthetics of the posterior torso led to innovation with J-torsoplasty and oblique flankplasty.

**Objectives:**

The goal of this study was to demonstrate in a large clinical series and in a range of case presentations from 2 plastic surgeons that oblique flankplasty with lipoabdominoplasty (OFLA) optimally narrows the waist, suspends lateral buttocks and thighs, and integrates with J-torsoplasty and vertical thighplasty to tighten skin and aesthetically contour the torso and thighs with an acceptable rate of complications.

**Methods:**

Retrospective chart review of 151 consecutive flankplasties between June 2010 and April 2023, including sex, age, BMI, associated operations, complications, and revisions was performed. Five case presentations were accompanied by limited photographs and a marking video.

**Results:**

Across a broad clinical spectrum, malleable oblique flankplasty resected bulging flanks and, facilitated by neighboring liposuction and/or J-torsoplasty, consistently pulled in lax skin and anchored through cadaver-proven dense dermal adherences lax tissues to create a long-lasting skintight shapely torso and upper thighs, with only 3.3% problematic wounds. Five diverse cases showed broad applicability.

**Conclusions:**

OFLA, often with J-torsoplasty and neighboring liposuction, aesthetically recontours torso skin laxity in a variety of presentations with a low rate of complications in a high-risk population.

**Level of Evidence: 3:**

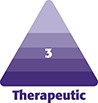

Optimizing treatment of body dysmorphia after massive weight loss (MWL) is formidable. Reversal of objectionable body contours with skin laxity necessitates coordinated artistic surgery, often beset by surgical, medical, financial, and safety constraints. Aesthetic improvements of the breast, chest, abdomen, buttocks, back, and upper thighs need to be harmonious, with all transitions and gender-specific contours addressed in as few stages as safely possible. Conceived by senior author D.J.H. in 1998, total body lift surgery (TBL) emphasized comprehensive skin tightening, leaving improved aesthetic contours through circumferential transverse upper and lower body resections, augmented as needed with deepithelialized neighboring flaps of excess tissue.^1,2^

During his initial 12-year TBL transverse excision practice, D.J.H. often noticed, and some patients complained of, poor posterior aesthetics. The upper body lift (UBL) bra line scar may pigment, widen, and constrict, while bordered by skin rolls. Lower body lift (LBL) leaves bulging flanks and a lengthened midline gluteal cleft, and within 6 months often settles with flat central buttocks and lateral hip prominences, depressed lateral gluteal regions, a circumferential constricted scar, and saddlebags. This so called LBL deformity is not so much a manifestation of damaged connective tissue after MWL but rather a by-product of ignoring bulging flanks and horizontal skin laxity, followed by inadequate soft tissue anchoring of a tight closure along the lateral pelvic brim.

In an exemplary post-MWL case published by D.J.H. in 2013, and up to today by others, LBL deformity has been ignored (Figure 1).^3^ Compounding these aesthetic failures is a high rate of delayed healing due to tight closures of undermined flaps over bony prominences. Bony pressure and sheer forces are confounding etiologies. Complication rates up to 50% and poor appearance in contrast to a high rate of patient satisfaction led one meta-analysis to conclude that LBL and belt lipectomy were functional and not aesthetic.^4^

**Figure 1.**
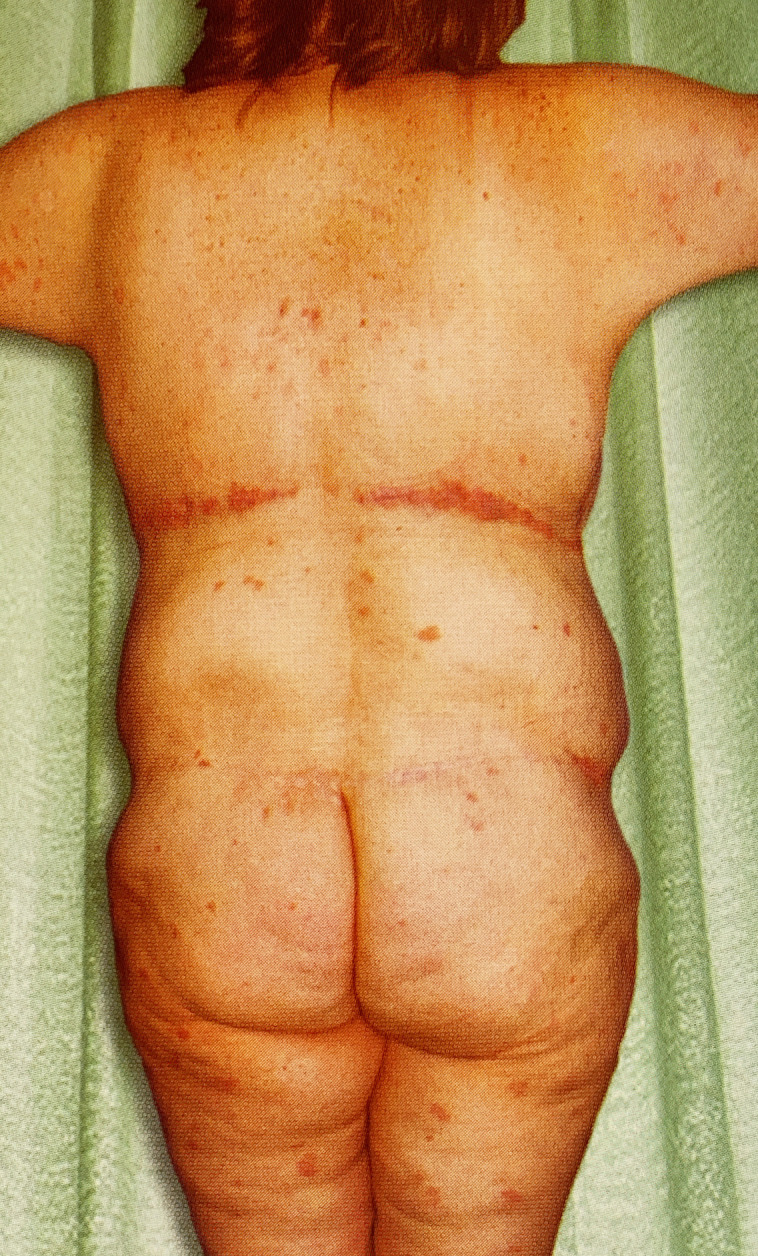
Unrecognized lower body lift deformity in D.J.H. book published in 2013. This 32-year-old MWL female had first-stage LBL with thighplasty and second-stage UBL with spiral flap breast reshaping and brachioplasty. She presented as an exemplary result, but not mentioned were her boxy torso, bulging lateral chest and flanks, flattened hips and buttocks, lateral gluteal depressions, constricting transverse scars, elongated midline gluteal cleft, and saddlebags. Reproduced with permission from Hurwitz DJ. Chapter 8. Sequencing and Timing of Surgery of the Post Bariatric Patient. *Cosmetic Surgery After Massive Weight Loss.* Thaller SR and Mimmis Cohen M, eds. JP Medical Ltd. 2013, page 70. LBL, lower body lift; MWL, massive weight loss; UBL, upper body lift.

Seeking improved posterior aesthetics and inspired by the horizontal as well as vertical skin excess removal of the boomerang pattern correction of gynecomastia, D.J.H. innovated with 2 oblique posterior torso excision patterns.^5^ First, J-torsoplasty UBL removes tissue vertically along the lateral chest, obliquely across the posterior chest and horizontally across the lower anterior chest.^6^ The isolated skin is excised or deepithelialized for a buried trans–anterior serratus perforator spiral flap breast reshaping. The closure lies inconspicuously along the lateral chest and inframammary folds. Second, oblique flankplasty LBL excises all flank redundancy and upon closure centripetally tightens and contours the mid torso and raises the lateral buttocks and upper lateral thighs. Oblique flankplasty along with lipoabdominoplasty (OFLA) was first performed on males in 2010 and then females in 2014.^7^ After a rapid and secure 2-layer running barbed suture closure, OFLA deeply tapered the waist, tightened the abdomen, reduced mid-back rolls, defined small hips, rounded lateral buttocks, and effaced saddlebags without raising the midline gluteal crease.^8^

The superiority of oblique flankplasty for both correction of flank deformity and long-lasting improved posterior torso aesthetics was confirmed through grading of standardized images by 12 plastic surgery residents.^9^ Despite higher Pittsburgh deformity ratings, the initial 36 OFLA patients were significantly improved and more aesthetically appealing then 46 randomly selected patients with previous circumferential LBLs.

Well before the oblique flankplasty, D.J.H. published objections to the fleur-de-lis abdominoplasty (FDL).^10^ Beyond an undesirable full-length midline abdominal scar, FDL creates a flat, featureless central abdomen with minimal narrowing of the waist and is beset with a high incidence of suprapubic and umbilical delayed healing.^11,12^ Because OFLA transversely tightens all but the most severe abdominal horizontal skin excess, FDL is now limited to revision of unsightly midline abdominal scars during closure of wide diastasis recti or central hernias, or patient refusal of flankplasty.

Since October 2020, plastic surgeon associate and coauthor A.A.D. contributed additional insights and adaptations. This combined update quadrupled the number of cases while maintaining outcome quality.

## METHODS

Candidates for oblique flankplasty were in good physical and mental health, with sagging skin of the body, desired a narrower and tighter waist, and accepted a posterior scar above the underwear. Patients with morbid obesity, inadequately controlled metabolic disease, anemia, poorly controlled hypertension, cigarette smoking, or unreasonable expectations or body dysmorphic syndrome were excluded. Preoperative nutritional assessment with dietary adjustments was instituted as needed. Patients with coagulopathy and a history of deep vein thrombosis or pulmonary embolism were prophylactically managed with a week of postoperative anticoagulant chemoprophylaxis and portable sequential compression stockings for 1 month.

Patients learned that LBL was the standard approach to treat lower posterior torso tissue laxity. Guided by pinch approximation, a transverse excision of loose skin reduced vertical skin excess and under a high tension suture closure beneath underwear lifted the buttocks and lateral thighs. They understood that oblique flankplasty with above-underwear low back scars was an alternative to LBL. Preoperative consent included permission to be deidentified and to use their clinical photographs and analyze their data for inclusion in a report, consistent with the guidelines of the Helsinki Accord of 1975.

One hundred and fifty-one (151) consecutive flankplasty charts were reviewed between June 2010 and April 2023, including sex, age, BMI, associated operations, complications, revisions, photographs, and videos.

### OFLA Markings

#### Case 1

**Figure 2.**
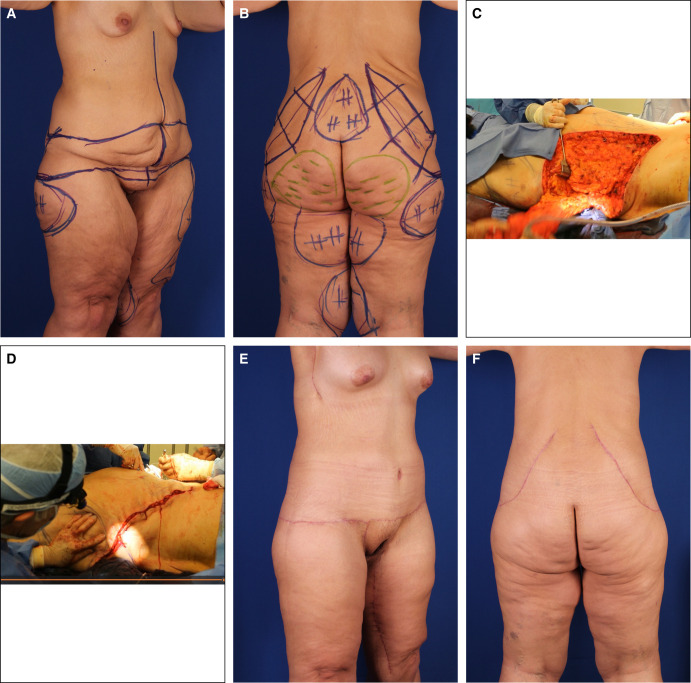
Case 1. (A) Right anterior oblique and (B) posterior preoperative views of 37-year-old, 6′ 1″, 230-pound (BMI 28) female after MWL, with bulky loose-skinned torso and thighs and small, constricted breasts. She is marked for OFLA, VASERlipo, and lipoaugmentation of the buttocks. The transverse lines of her lipoabdominoplasty continue into the oblique flankplasty and are lowered to meet her saddlebags. Obliquely oriented flank excisions are surrounded by pluses for liposuction and minuses for buttock lipoaugmentation. (C) The patient is prone with her head to the right. Her large right flank resection to the lumbodorsal fascia is ready for lateral amputation. (D) With most of the flankplasty closed, the surgeon is approximating the lateral wound by pulling the no. 2 barbed suture while pushing the lateral thigh. (E) Right anterior oblique and (F) posterior views 6 months after OFLA and 4 months after vertical medial thighplasty with pubic monsplasty, extended L-brachioplasty, and submuscular 450-cc breast gel implant augmentation. She has rounded breast enlargement, a flat and tight-skinned abdomen, and absent back rolls with smoothly recessed flanks. Her thighs are smaller, tighter, and conical with no saddlebags. BMI, body mass index; OFLA, oblique flankplasty with lipoabdominoplasty; MWL, massive weight loss.

After drawing a vertical midline from the xyphoid to the mons pubis, a conservative estimate of the width of lower abdominal resection was drawn transversely (Figure 2A). Facing the lateral torso with the lateral buttocks and thigh maximally pushed upward, we marked the lateral width of the flank/abdominoplasty excisions as determined by pinch gathering of tissues inferior to the abdominoplasty extended upper incision line. For a saddlebag deformity, the lower incision was drawn just superior to the bulge, and then the width of resection was similarly determined. The same maneuver was performed on the opposite side.

Facing the posterior torso, we drew obliquely oriented lines along the flank crests (Figure 2B). Midway, we did a pinch gather of the excess low back tissue to mark the width of the flank resection, equally on each side of that meridian. The superior incision was drawn from about the paraspinous twelfth rib, across the superior middle mark to the abdominoplasty superior line. Again, from the paraspinous twelfth rib, a vertical inferior incision line was dropped through the inferior middle mark to continue to the abdominoplasty inferior line. We did a segmental pinch grab along the excision to confirm the resection width. The opposite side was similarly drawn. Differences in resection widths and orientation were reconciled to achieve symmetrical results.

Usually, a hockey stick–like hemiellipse was drawn, with the paraspinous portion being the handle. Upon closure, the shorter superior incision line was advanced posteriorly. For symmetrical simultaneous closures, the upper and lower incision lines were aligned. Starting at the superior apex, 4 hatch marks were made at 10-cm intervals. Neighboring areas were marked for liposuction and lipoaugmentation.

**Figure 3.**
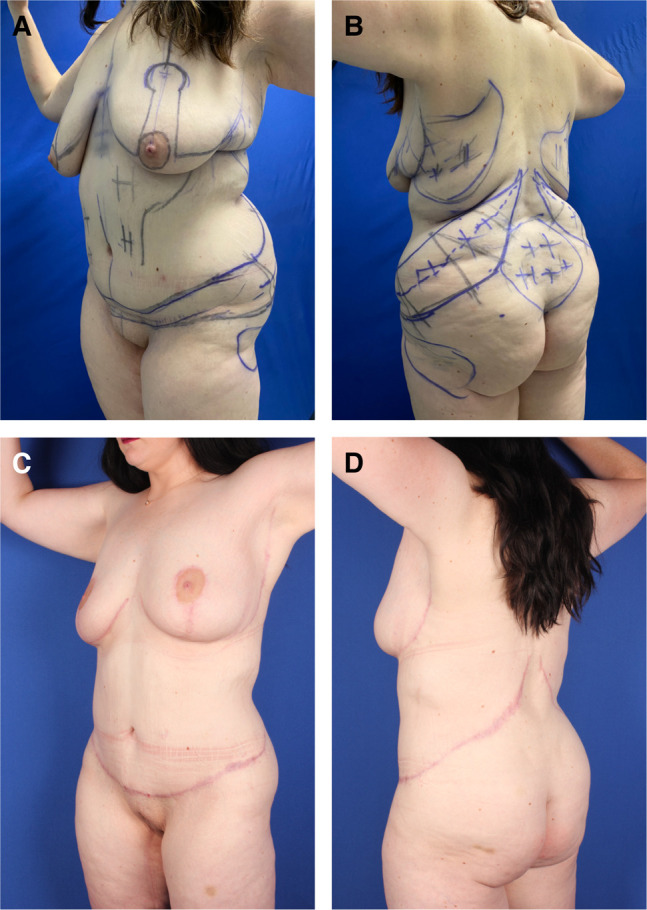
Case 2. (A) Left anterior and (B) posterior oblique preoperative views of an obese, BMI 31.6, 27-year-old female with ptotic, low breasts, multiple large back rolls, and sagging abdomen after 30-pound weight loss. She is marked for OFLA, pluses for VASERlipo, Wise pattern mastopexy, J-torsoplasty, and minuses for lipoaugmentation of lower lateral buttocks. Video shows the marking for OFLA. (C) Left anterior and (D) posterior oblique views 5 months later show fading oblique scars about tight and curvaceous breasts and torso, without rolls of adiposity. BMI, body mass index; OFLA, oblique flankplasty with lipoabdominoplasty.

### Operation

The patient was turned prone onto 2 silicone gel rolls transversely placed under the anterior costal margins and upper thighs, which assisted with ventilation and unimpeded posterior advancement of abdominal skin during closure of the flank excision sites. After liposuction, the inferior flank marking was incised. Proceeding perpendicularly through superficial subcutaneous tissue, well-defined Scarpa's fascia was incised to globular buttock adipose tissue. Depending on artistic judgement, the surgeon could either skim over this buttock fat along the subcutaneous fascial plane to preserve it for volume or continue the incision directly through the globular buttock fat to the pelvic rim to reduce the upper buttock volume. The dissection proceeded until muscular fascia was exposed. With towel clamps along the non-undermined inferior flap, we pulled the lateral buttocks to the previously drawn superior incision to confirm the resection width. The superior lines were incised through subcutaneous tissue to the latissimus dorsi and external oblique fascia. With the perimeter incision completed, we resected the flanks from the medial back to the lateral extent of the abdominoplasty. (Figure 2C).

Starting in the middle, with a double-armed no. 2 PDS barbed suture, we widely encompassed Scarpa's fascia, with a third bite including muscular fascia near the upper incision^8^ Without undermining but assisted with an upward push on the buttock and thigh, we slid the buttock skin and subcutaneous fascia over the gluteal adipose for a smooth layered advancement of buttocks to the depths of the lumbodorsal fascia and external obliques (Figure 2D). Complete closure was performed with intradermal 2-0 Monoderm (Quill; Corza Medical, Westwood, MA). We covered the patient with a sterile gown and turned the patient supine for lipoabdominoplasty.

At 6 months, OFLA was followed by vertical medial thighplasty, which provided a deepened waist, flat abdomen, and smaller conical thighs without saddlebags (Figure 2E, F). Two years later, this out-of-town patient reported a stable result.

**Figure 4.**
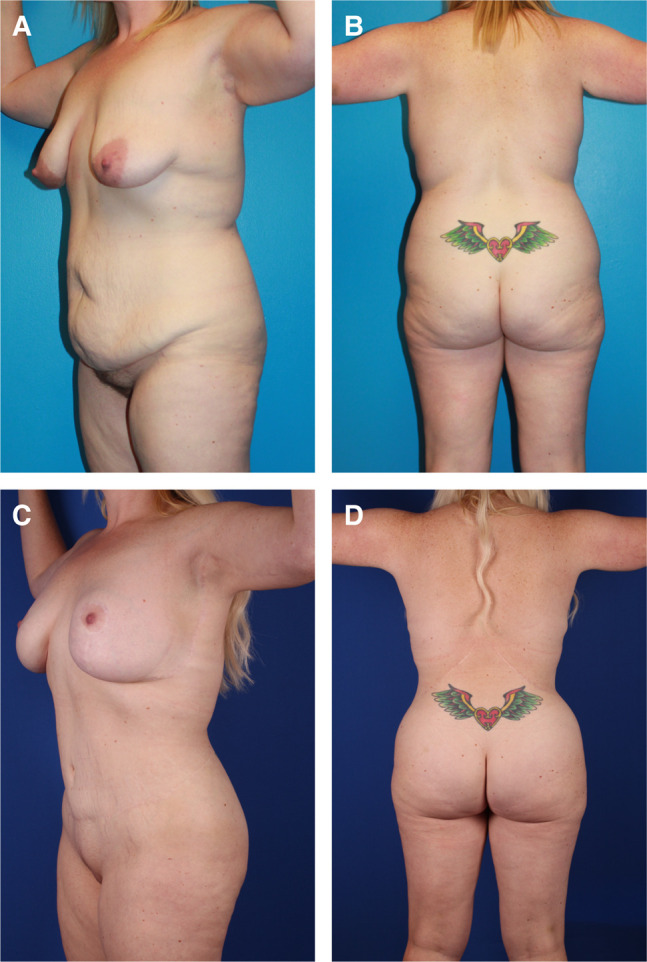
Case 3. (A) Left anterior oblique and (B) posterior views of a 43-year-old normal-weight female after 80-pound weight loss reveal sagging full abdomen, small ptotic breasts, prominent lateral chest rolls, scapula bulges, overfilled flanks merging into oversized hips with depressed lateral gluteal regions and buttocks sagging into saddlebags. (C) Left anterior oblique and (D) posterior views are 6 years after OFLA with lateral gluteal lipoaugmentation followed by J-torsoplasty with spiral flap breast reshaping and L-brachioplasty. Her tight skin and contour improvement persists with imperceptible scars. Her narrowed waist smoothly transitions to a tapering chest and rounded buttocks without elongation of the midline crack. OFLA, oblique flankplasty with lipoabdominoplasty.

**Figure 5.**
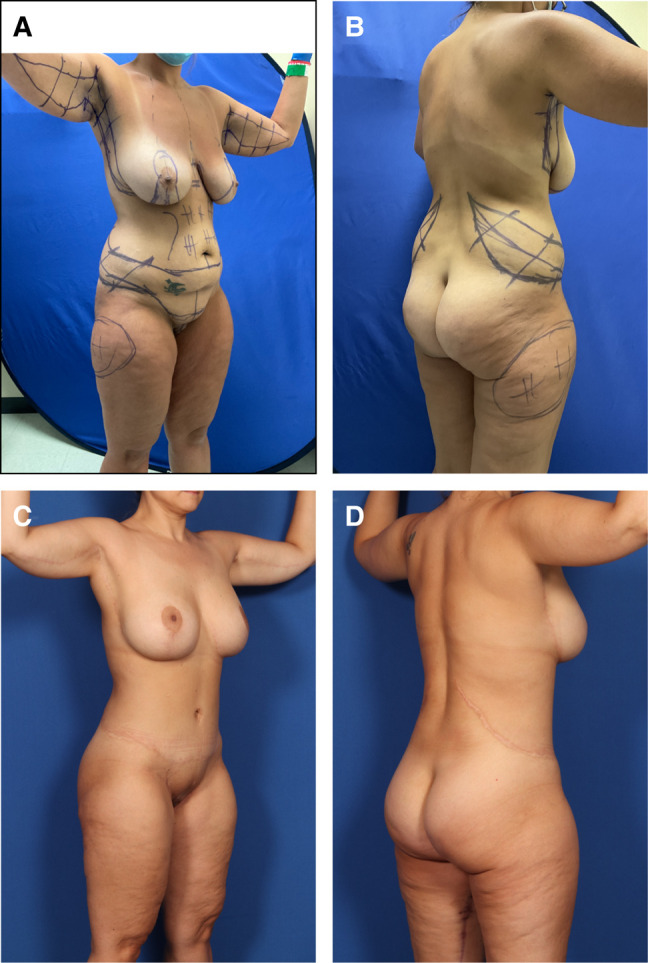
Case 4. (A) Right anterior oblique and B) posterior views of a 34-year-old low BMI (23.6) 42-year-old female after losing 80 pounds who requests single-stage TBL for enlarged sagging breasts, pannus, mons pubis, flanks, breasts, arms, and thighs. She is marked for an L-brachioplasty, J-torsoplasty with spiral flap reshaping of her mastopexy, and OFLA, as well as liposuction of her saddlebags. (C) Right anterior oblique and (D) posterior oblique views 1 year later and 6 months after an L-thighplasty. She has curvaceous tight skinned narrower arms, thighs, and torso, with full projecting rounded breasts and buttocks and absent saddlebags. BMI, body mass index; OFLA, oblique flankplasty with lipoabdominoplasty; TBL, total body lift.

**Figure 6.**
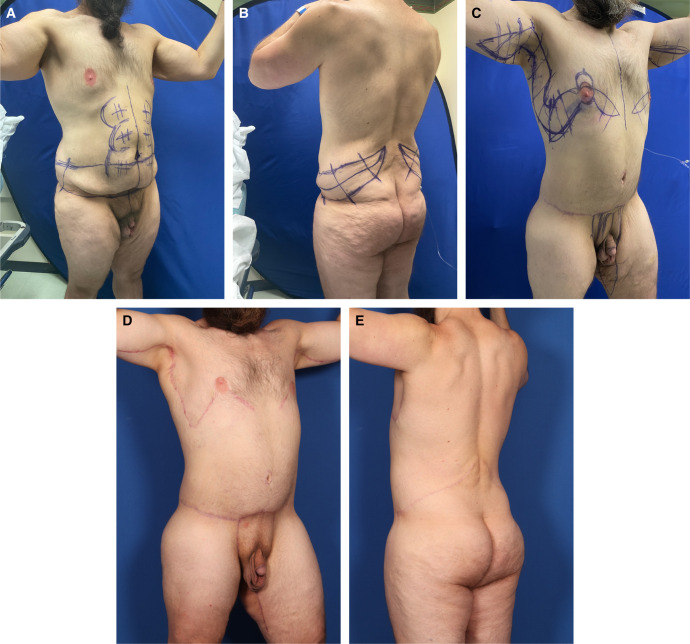
Case 5. (A) Right anterior oblique and (B) left posterior oblique views of an overweight 42-year-old 6′ 2″ male with 150-pound weight loss (400 to 250 pounds, BMI 32.2) show broad gynecomastia with lateral chest lipodystrophy, a distinct inframammary fold, and oversized elongated NACs sagging below the inferior pectoralis major border. Upper arms, protuberant abdomen, mons pubis, and proximal thighs are ptotic. An upper transverse gluteal linear depression is crossed by an elongated midline gluteal crack. He is marked for high-definition liposuction as part of a TULUA, which extends posteriorly into an oblique flankplasty. (C) Right anterior oblique view 6 months after his OFLA shows a tight-skinned well-contoured lower torso. The markings are for a picture frame monsplasty, limited L-thighplasty, and limited L-brachioplasty, extended by a J-torsoplasty into a boomerang excision pattern correction of gynecomastia. (D) Right anterior oblique and (E) left posterior oblique views 5 months after the second stage show a tight-skinned muscular masculine torso and aesthetically reduced upper arms and thighs with short scars. The smoother, rounder, tight buttocks have a shorter midline crack. His gynecomastia is corrected, with a smaller NAC raised to the lower pectoralis muscle and obliteration of the IMF. BMI, body mass index; IMF, inframammary fold; NAC, nipple-areola complex; OFLA, oblique flankplasty with lipoabdominoplasty; TULUA, transverse plication, undermining halted at umbilicus, liposuction, umbilicoplasty with skin graft, and abdominoplasty with low transverse scar localization.

OFLA might be immediately followed by a J-torsoplasty or a transverse UBL and breast reshaping, as shown in the Video and seen in Figures 3, 5. Usually, those operations were followed by a second stage at least 3 months later with or without brachioplasty and thighplasty, as seen in Figures 2, 4, 6.

## RESULTS

Over 13 years, 151 oblique flankplasties were performed in 35 males (23.2%) and 116 females (76.8%). The average age was 45.4 years (range 19-78) with an average BMI 28.3 (19.6-40.8) and weight lost averaging 114.9 pounds (12-246). Length of follow-up averaged about 1 year but could be as short as 2 months, often in the case of out-of-town patients, and as long as 10 years. Demographics and results are summarized in Table.

**Table 1.** Summary of Patient Demographics and Results

	Average or no. of patients	Range or percentage
Average age (years)	45.4	19–78
Average BMI	28.3	19.6–40.8
Average weight lost (lb.)	114.9	12–346
Weight loss method		
Gastric bypass	35	23.2%
Gastric sleeve	20	13.2%
Diet/exercise	54	35.8%
None/unknown	42	27.8%
Total cases	151	
Male	35	23.2%
Female	116	76.8%
Inpatient	24	15.9%
Outpatient	127	84.1%
Pittsburgh grade 1	14	9.3%
Pittsburgh grade 2	36	23.8%
Pittsburgh grade 3	101	66.9%
Concurrent procedures		
Abdominoplasty	107	70.9%
Limited Abdominoplasty	14	9.3%
Fleur-de-lis Abdominoplasty	6	4.0%
Buttock fat transfer	12	7.9%
Thighplasty	21	13.9%
Panniculectomy	9	6.0%
Mastopexy/upper body lift	21	13.9%
Other	16	10.6%
Complications		
Minor dehiscence (3 cm)	5	3.3%
Seroma	15	9.9%
Cellulitis/infection	12	7.9%
Revision/return to operating room	4	2.6%

BMI, body mass index.

Oblique flankplasty was not performed as an isolated procedure. Most flankplasties were performed with lipoabdominoplasty (70.9%), followed by thighplasty (13.9%). Indications for the procedure were commonly a Pittsburgh Scale grade 3 flank or back (66.9%) with weight loss primarily from calorie restriction (35.8%) and an average weight loss of 115 pounds. The majority were outpatient (84.1%), with 1 unplanned hospital admission for transfusion after multiple procedures and 1 for pain management.

The new procedures are safer than LBL, with notable reduction in major wound healing complications. Overall seromas occurred in 9.9% of patients, and often coincided with subsequent minor infections or cellulitis (7.9%). Rarely did these seromas, wounds, or infections require return to the operating room (2.6%), with the majority managed with aspiration and oral antibiotics. Small wound healing disturbances, scabbing, or superficial edge necrosis occurred at an expected rate (15.2%), but larger wounds requiring advanced care or secondary closure were rare (3.3%).

All oblique flankplasty patients reported that their aesthetic improvement met expectations, and the back scars were either inconsequential or at least better than traditional LBL, especially in lieu of the FDL abdominal vertical scar. No patient requested flankplasty revision. In addition to demonstrative Case 1, the 4 following case presentations represent the clinical spectrum, from obese through thin female and 1 male.

### Clinical Case Examples

#### Case 2

The markings in an obese patient with previous abdominoplasty and without saddlebag deformity are shown in the Video and Figure 3. The lateral extent of the flankplasty lay just inferior to the iliac crest, and neighboring liposuction was extensive. This obese female with skin laxity was a 27-year-old with BMI 32, who lost 140 pounds after bypass but regained 40 pounds after her abdominoplasty. Concerned about her sagging breasts, back rolls, and sagging abdomen, she requested single-stage TBL, consisting of 3000-mL VASERlipo (Solta Medical, Bothell, WA), 400-mL buttock lipoaugmentation, OFLA, J-torsoplasty with Wise pattern mastopexy and spiral flap reshaping (Video, Figure 3). Five months later, with fading scars, she liked her raised and rounded breasts, with a curvaceous figure and tight skin.

#### Case 3

This overweight female with skin laxity was 37 years old, 5′ 7″, 173 pounds, BMI 26.6. She lost 130 pounds after gastric bypass and then gained back 50 pounds with pregnancy and requested a staged TBL (Figure 4). Six years after her OFLA and 5 years after her J-torsoplasty with spiral flap breast reshaping and L-brachioplasty, she remained pleased with her stable curvaceous body with imperceptible scars.

#### Case 4

The female patient with normal weight and lax skin was 5′ 3″, 133 pounds, BMI 23.5. She disliked her deflated breasts, bulging flanks, full lower abdomen, saddlebag deformity, hanging arms, and loose thighs. She desired a single-stage OFLA and spiral flap breast reshaping with J-torsoplasty (Figure 5). After her 5-hour outpatient operation, she healed well. Six months later, she returned for a vertical thighplasty. One year after stage 1, she had a curvaceous figure with well-shaped breasts, buttocks, upper arms, and thighs, without saddlebags or conspicuous scars.

#### Case 5

The patient was a 41-year-old male, 6′ 2″, BMI 32.2, with weight loss of 220 pounds, followed by regaining 60 pounds, who complained of gynecomastia and a bulging feminine lower body. Stage 1 was a combined TULUA and oblique flankplasty (Figure 6). After uneventful healing, 7 months later he underwent J-torsoplasty, boomerang correction of severe gynecomastia, limited L-brachioplasty, and vertical thighplasty with pubic monsplasty. One year after stage 1, he was pleased with his masculine appearance, which included gynecomastia correction, a tight-skinned dominant upper body, narrow waist, and short rounded buttocks. Once his scars have completely faded his lower NAC excess will be excised under local anesthesia.

## DISCUSSION

This retrospective 151 case, 2-surgeon clinical update confirms that oblique flankplasty suffers few complications and consistently meets aesthetic goals. With adaptability to the full range of loose skin deformity and lipodystrophy across all treatable weight losses, BMIs, and ages, the indications for OFLA have expanded. The OFLA reliably and aesthetically lifts, shapes, and smoothly transitions backs, buttocks, lateral thighs, flanks, hips, and abdomens. Moreover, OFLA compliments both transverse and J-torsoplasty UBL.

In retrospect, luminaries Lockwood, Teitelbaum, and Ali anticipated OFLA. Lockwood's lateral high-tension abdominoplasty improved abdominal appearance through a superior lateral resection of excess tissue.^13^ Praising its reduction of transverse epigastric laxity and suprapubic tension, Teitelbaum later endorsed a broader superior lateral abdominoplasty excision.^14^ Belt lipectomy coursed superior to LBL, along the hip/flank junction. While exerting a slight posterior pull on the anterior abdomen, it neither deepened the flanks nor corrected saddlebag deformity.^15^

For optimal vertical epigastric contours, we rely on our modified central high-tension abdominoplasty by tightly suturing 3 small deepithelialized epigastric flaps to rectus fascia at the base of the umbilicus. Between this umbilicoplasty and the OFLA, a thinned abdominal skin flap is tightly wrapped and contoured.^16,17^

With no tension on the central buttocks, oblique flankplasty does not flatten them, so it generally obviates problematic auto augmentation.^18^ Instead, limited lipoaugmentation has been performed as needed in about 10% of cases, with the flank as the donor excision site. With medial rotation of the lateral buttock flap, buttocks were roundly shaped and fat recipient sites were limited to previous depressions and cellulite.

Because the oblique flank closures were not stressed by abdominal flexion, nor subjected to direct pressure or sheering, major wounds were under 5%. Our minor OFLA incisional wound rate was around 15%, lower than the reported 25.2%.^19^ If the OFLA has delayed healing, it is usually anterior, suprapubic or along the anterior iliac crests, requiring only examination room debridement and weeks of dressing care.

With variable retention of premuscular adipose, desired gender characteristics can be emphasized. Except in obese patients, deep gluteal adipose is generally preserved in females, whereas this globular adipose is usually resected in males. Oblique flankplasty with TULUA in overweight males and gender reassignment epitomizes these options.^20^

While flank closures are generally minimally painful due to the dermatomal excision pattern, we achieved up to 4 days pain free following OFLA with long-acting circumferential nerve blocks under the easily palpable proximal eleventh and twelfth ribs. Excess swelling of the closure, ameliorated by early postoperative Hivamat (Physiomed, Georgetown, TN) lymphatic massage, is usually an issue with nearby large-volume VASERlipo and radiofrequency subcutaneous tissue tightening. The early scars tend to be slightly raised and red, but flatten over several months and may fade completely, especially with pale skin. This optimal scar maturation is due to consistent primary healing and the orientation of closure.^21-23^

Due to the oblique flankplasty's many advantages, we only consider LBL for (1) oversized sagging buttocks and lateral thighs with small flanks, (2) gluteal flap harvest for buttock augmentation, or (3) patient refusal of flankplasty.

This retrospective clinical review of 2 surgeons’ sequential collective experience with oblique flankplasty is contemporaneously uncontrolled and suffers the limitations of level 4 evidence, with the vagaries and insufficient documentation of clinical practice. We have confirmed the positive outcomes of our pilot study, supported by independent clinical evaluators and coauthors.^9^ Within the limits imposed by the musculoskeletal form and obesity, all oblique flankplasty cases obtained near optimal contouring and skin tightness. While innovator D.J.H. is understandably biased, coauthor flankplasty-naive and critical plastic surgeon A.A.D. joined the practice just 3 years ago. Plastic surgeon colleagues, also instructed by D.J.H., have shared similar positive experiences.^24^

## CONCLUSIONS

Consistent clinical experience confirms that OFLA, usually with neighboring liposuction and/or J-torsoplasty, broadly and aesthetically recontours and corrects torso laxity with a low rate of complications. Due to the ease of execution, improved outcomes, and consistent patient satisfaction, OFLA has become our preferred circumferential LBL, despite leaving oblique lumbar scars. Through posterior correction of horizontal excess upper abdominal skin, OFLA obviates FDL abdominoplasty. Moreover, the quest to avoid lateral buttock sag and recurrent saddlebag deformity has been solved.^25^ The 2004 aesthetic imperative of TBL is finally being realized.

## References

[1] Hurwitz DJ. Single-staged total body lift after massive weight loss. Ann Plast Surg. 2004;52(5):435–441. doi: 10.1097/01.sap.0000123361.14654.a5 15096919

[2] Hurwitz DJ, Agha-Mohammadi S, Ota K, Unadkat J. A clinical review of total body lift surgery. Aesthet Surg J. 2008;28(3):294–305. doi: 10.1016/j.asj.2008.03.001 19083540

[3] Hurwitz DJ. Keys to success in chapter 8 sequencing and timing of surgery of the post bariatric patient. In: Thaller SR and Mimis C, eds. Cosmetic Surgery After Massive Weight Loss, Medical Publishers; 2013:57–72.

[4] Carloni R, De Runz A, Chaput B, et al Circumferential contouring of the lower trunk: indications, operative techniques, and outcomes-a systematic review. Aesthetic Plast Surg. 2016;40(5):669. doi: 10.1007/s00266-016-0660-7 27439535

[5] Hurwitz DJ. Boomerang pattern correction of gynecomastia. Plast Reconstr Surg. 2015;135(2):433–436. doi: 10.1097/PRS.0000000000000933 25626790

[6] Clavijo-Alvarez JA, Hurwitz DJ. J torsoplasty: a novel approach to avoid circumferential scars of the upper body lift. Plast Reconstr Surg. 2012;130(2):382e–383e. doi: 10.1097/PRS.0b013e31825903e5 22842449

[7] Hurwitz D Chapter 4. 1 background: enhancing female features In: Comprehensive Body Contouring: Theory and Practice, Springer, New York, 2016:63–69. doi 10107/978-3-662.

[8] Hurwitz DJ, Reuben B. Quill barbed sutures in body contouring surgery: a 6-year comparison with running absorbable braided sutures. Aesthet Surg J. 2013;33(3 Suppl):44S–56S. doi: 10.1177/1090820X13498506 24084879

[9] Hurwitz DJ, Beidas O, Wright L. Reshaping the oversized waist through oblique flankplasty with lipoabdominoplasty. Plast Reconstr Surg. 2019;143(5):960e–972e. doi: 10.1097/PRS.0000000000005574 30807493

[10] Hurwitz D. Optimizing body contour in massive weight loss patients: the modified vertical abdominoplasty. Plast Reconstr Surg. 2004;114(7):1917–1923. doi: 10.1097/01.PRS.0000142998.57916.E3 15577368

[11] Costa LF, Landecker A, Manta AM. Optimizing body contour in massive weight loss patients: the modified vertical abdominoplasty. Plast Reconstr Surg. 2004;114(7):1917–1926. doi: 10.1097/01.prs.0000142997.63346.95 15577368

[12] Friedman T, O'Brien Coon D, Michaels VJ, et al Fleur-de-Lis abdominoplasty: a safe alternative to traditional abdominoplasty for the massive weight loss patient. Plast Reconstr Surg. 2010;125(5):1525–1535. doi: 10.1097/PRS.0b013e3181d6e7e0 20145584

[13] Lockwood T. High-lateral-tension abdominoplasty with superficial fascial system suspension. Plast Reconstr Surg. 1995;96(3):603–615. doi: 10.1097/00006534-199509000-00012 7638284

[14] Teitelbaum S. Demystifying high-lateral-tension abdominoplasty. Aesthet Surg J. 2006;26(3):325–329. doi: 10.1016/j.asj.2006.03.006 19338916

[15] Aly AS, Cram AE, Chao M, Pang J, McKeon M. Belt lipectomy for circumferential truncal excess: the university of Iowa experience. Plast Reconstr Surg. 2003;111(1):398–413. doi: 10.1097/01.PRS.0000037873.49035.2A 12496613

[16] Le Louarn C, Pascal JF. High superior tension abdominoplasty. Aesthetic Plast Surg. 2000;24(5):375–381. doi: 10.1007/s002660010061 11084700

[17] Hurwitz DJ. Invited discussion on: simplified technique for creating an umbilicus with scarless caudal aspect and superior hooding. Aesthetic Plast Surg. 2022;46(3):1290–1292. doi: 10.1007/s00266-021-02724-3 35091772

[18] Coon D, Gusenoff JA, Kannan N, El Khoudary SR, Naghshineh N, Rubin JP. Body mass and surgical complications in the postbariatric reconstructive patient: analysis of 511 cases. Ann Surg. 2009;249(3):397–401. doi: 10.1097/SLA.0b013e318196d0c6 19247025

[19] Winocour J, Gupta V, Ramirez JR, Shack RB, Grotting JC, Higdon KK. Abdominoplasty: risk factors, complication rates, and safety of combined procedures. Plast Reconstr Surg. 2015;136(5):597e–606e. doi: 10.1097/PRS.0000000000001700 26505716

[20] Babaitis R, Villegas FJ, Hoyos AE, Perez M, Mogollon IR. TULUA Male high-definition abdominoplasty. Plast Reconstr Surg. 2022;149(1):96–104. doi: 10.1097/PRS.0000000000008680 34936608

[21] Wilhemi BT, Blackwell SJ, Phillips LG. Langer's lines: to use or not to use. Plast Reconstr Surg. 1999;104(1):208–214. doi: 10.1097/00006534-199907000-00033 10597698

[22] KRAISSL CJ. The selection of appropriate lines for elective surgical incisions. Plast Reconstr Surg (1946). 1951;8(1):1–28. doi: 10.1097/00006534-195107000-00001 14864071

[23] Lemperle G, Tenenhaus M, Knapp D, Lemperle SM. The direction of optimal skin incisions derived from striae distensae. Plast Reconstr Surg. 2014;134(6):1424–1434. doi: 10.1097/01.prs.0000438462.13840.21 25415105

[24] Personal communication Drs., Mohammed Afifi, Jeffrey Gusenoff. Guy Stoffman, Omar Beidas, Soren Konneker, and Donna Tepper.

[25] Hurwitz DJ, Rubin JP, Risin M, Sajjadian A, Sereika S. Correcting the saddlebag deformity in the massive weight loss patient. Plast Reconstr Surg. 2004;114(5):1313–1325. doi: 10.1097/01.prs.0000135862.83833.5f 15457056

